# The Cyclopædia of Practical Surgery

**Published:** 1838-07

**Authors:** 


### Art. V.

1. *The Cyclopædia of Practical Surgery.* Edited by William B. Costello, m.d. Part I. — *London, April,* 1837. 8vo. — *Article,* Abortion, by Dr. Ryan.
2. *The Cyclopædia of Practical Medicine and Surgery.* Edited by Isaac Hays, m.d. Part I.—*Philadelphia, July,* 1833. 8vo.—*Art.* Abortion, by Dr. Dewees.
3. *A Dictionary of Practical Medicine, &c.* By James Copland, m.d. &c. Part I.—*London,* 1832. 8vo.—*Art.* Abortion, by Dr. Copland.
4. *The Cyclopædia of Practical Medicine.* Edited by John Forbes, m.d. f.r.s., Alexander Tweedie, m.d., and John Conolly, m.d. Part I.—8vo. *London, January,* 1832.—*Art.* Abortion, by Dr. Lee.
5. *Dictionaire de Médecine et de Chirurgie pratiques.* Par MM. Andral, Begin, Blandin, Bouillaud, &c. &c.

*Dictionary of Practical Medicine and Surgery, &c.* Vol. III.—*Paris,* 1829. 8vo. *Art.* Avortement, by Prof. Dugès.

6. *Encyclopädisches Wörterbuch der medicinischen Wissenschaften. Herausgegeben, von den Professoren der medicinischen Facultät zu Berlin,* C. F. v. Graefe, C. W. Hufeland, H. F. Link, K. A. Rudolphi, E. v. Siebold.

*Encyclopædic Dictionary of Medical Science, &c.* Vol. I.—*Berlin,* 1828. 8vo.—*Art.* Abortus, by Dr. von Siebold.

On the appearance of Mr. Costello's Cyclopædia, we were led to compare some of its articles with those on the same subjects in other works of the kind; and it occurred to us that we might be able to lay before our readers some valuable practical articles founded on a critical survey of the whole of the publications on the same plan, recently published. The delay that has taken place in the publication of the Cyclopædia of Surgery has prevented us from carrying our plan into execution; but the present article affords a specimen of the information which it is capable of supplying. We have fixed on the subject of Abortion as the most important that occurs in the alphabetical arrangement followed in these works.

Although in the works before us the whole subject of premature expulsion is treated of under the general head of *Abortion*, yet, with the exception of Professor v. Siebold and Dr. Dewees, each writer has limited the strict meaning of this term to expulsions of the fœtus occurring before it has attained a sufficient degree of development to enable it to support an existence separate from the mother. Premature expulsion of the fœtus may therefore be considered under two distinct heads,—Abortion, and Premature Labour; it being only in the latter division that the child will be capable of maintaining an independent existence. The precise period at which the one division terminates and the other commences has been variously given. Thus Dr. Copland and Professor Dugès consider that the term premature labour is not applicable till the fœtus has passed the sixth month of utero-gestation. Dr. Dewees, who has not founded his division of the subject into abortion and premature delivery on the capability or incapability of the fœtus to support an independent existence, which we are rather surprised at, fixes the limit of abortion to expulsions of the ovum before the fifth month. The German authors have usually divided premature expulsion into three periods, "*abortus*," "*partus immaturus*," and "*partus prematurus:*" under the first division being classed all those expulsions which take place during the first sixteen weeks of pregnancy; under the second all those occurring from this period till the twenty-eighth week; after which time until about the thirty-seventh week they come under the last division, viz. premature labour. This arrangement, with a very trifling deviation, has been followed by Professor von Siebold.

The *causes* of abortion have been variously arranged by different authors; indeed to bring them under a simple, intelligible, and practical division is, we hold, by no means an easy task. We prefer the arrangement of the German and French authors, and are glad to find ourselves supported in this respect by Dr. Copland, who, in quoting from the article of Professor Dugès, above alluded to, says, "these may be divided into such as act primarily upon the mother or depend upon her, and into those which are connected with the product of conception, and are owing to diseases of the fœtus or its appendages." Each of these main divisions, as Professor v. Siebold observes, may be separately considered under the predisposing or the exciting causes. Neither Dr. Dewees nor Dr. Lee has given any distinct classification of the causes of premature expulsion, although in justice we must add that this subject has been ably and concisely handled by both these gentlemen. Dr. Ryan's method of arrangement is not very intelligible; he however divides the predisposing causes into those which "affect the mother or the fœtus respectively."

Among the *predisposing causes* of premature expulsion we are surprised to find that Dr. Lee commences with the remark, that "abortion is a frequent occurrence in the early months of pregnancy, particularly among women of the lower classes of society who are exposed to much bodily fatigue and mental anxiety," because a very different result has usually been observed by those in extensive practice among the better classes, viz. that abortion is of much more frequent occurrence among females of the upper ranks, on account of their more delicate frame and greater susceptibility of external impressions, owing to a luxurious life, little exertion, &c.

"Rigidity of the uterine fibres, and an unyielding state of its parietes," have been mentioned as a cause of abortion by all the authors except Drs. Lee and Dewees; the former of whom makes the following just observation: "Those who have insisted on rigidity of the uterine fibres as a cause of abortion have been led into error, by supposing that the uterus enlarges during pregnancy by the mere force of the mechanical distension of the ovum, and not by the gradual development of all the textures of the organ in exact correspondence with the growth of the organs of the fœtus." (p. 11.) The causes of premature expulsion are very fully and ably considered by Dr. Copland. His eighth paragraph on the predisposing causes, "chiefly referrible to the *constitution and habit of the mother*," is very complete and well worthy of attention; we would willingly have quoted the whole of it had our limits permitted. His next paragraph (misprinted 10th), on "*the causes which depend upon the fœtus*," is equally good.

Dr. Lee has insisted considerably on the fact of a diseased condition of the ovum being one of the most frequent causes of abortion. Taking the word abortion in its strict meaning, viz. expulsion during the first twelve or sixteen weeks of pregnancy, we perfectly agree with him, and have the powerful authority of the celebrated Baer, (*see our first Number, p.* 240,) in confirmation that by far the majority of abortions are connected with an anormal state of the ovum. Dr. Copland has very properly noticed the important practical fact that the various causes of abortion act with increased energy and effect at those periods at which in the unimpregnated state the catamenia would have appeared. The same observation is made by M. Dugès: we might also mention Dr. Ryan, but as by far the greater part of his observations on the causes of abortion, with very trifling alterations, are taken from Dr. Copland, with the exception of three sentences from Dr. Lee and one from Dr. Dewees, it would be mere waste of time to notice them as original. Dr. Ryan in some places forgets what he has copied, and contradicts himself not a little now and then. Thus, at the commencement of page 15, he says, " women in the lower class of life, who are exposed to much bodily fatigue and mental emotions, are also very liable to this disease;" while at the latter part of the same page he observes, " it is to be recollected, that women in the lower ranks of life, who make great exertion and who are frequently exposed to falls and blows, very seldom miscarry."

In speaking of the connexion between the uterus and the placenta, we regret that Dr. Lee should have stated that " the placenta adheres to the uterus by means of the deciduous membrane alone, which is directly applied to the openings of the uterine sinuses." Although he was perhaps justified in making this observation at the time that the first Number of the Cyclopædia of Practical Medicine was published, from the slight degree of doubt which he had for a short time thrown upon the accuracy of the admirable researches on these subjects by the Hunters, yet, as the observations of these eminent anatomists have been most clearly and satisfactorily proved to be correct, the above-quoted sentence appears rather as a blot of inaccuracy upon an otherwise excellent and practical article. The author of the article in the Cyclopædia of Practical Surgery has adopted these views which we believe Dr. Lee himself now considers as incorrect; and in a somewhat diluted form has given the observations which conclude the paragraph above alluded to.

In examining the symptoms of abortion, as described in the different essays under examination, we regret that so little attention has been paid to a proper classification of them. It is true that Professors Siebold and Dugès, and Dr. Copland, to a certain extent, have virtually done so, but still not so as to excite the notice of the ordinary reader. The symptoms will, in chief measure, depend on the cause of the attack; hence the symptoms may be those produced by the previous death of the child, those arising from local injury or irritation, and those which depend on certain conditions of the whole system, more especially as regards the circulation. Without some attempt at arrangement we have a confused medley of symptoms, which, however correct, are nevertheless not so easily retained in the memory, and are sometimes not altogether intelligible to a beginner.

" The decided approach of abortion is indicated by two chief symptoms, viz. by the discharge of blood from the vagina, and uterine contractions." (*Dugès*, p. 669.) The value of these symptoms has been very variously stated by different authors, among whom none have given more plain and practical observations than Dr. Dewees: we regret not to have found something of the same sort in his present article. Professor Dugès considers that hemorrhage most frequently *precedes* the appearance of the pains; and, as far as respects distinct uterine contractions, we perfectly agree with him; but, on the other hand, a patient may suffer pain in the back and loins for some time without the appearance of hemorrhage and without abortion being a necessary result. Pains coming on at regular intervals like labour pains, being the result of uterine contractions, show that the uterus has already begun to act towards the ovum as a foreign body, and where this is the case expulsion is inevitable. On these grounds Dr. Burns has founded the following rule. "When uterine pain precedes or accompanies the discharge, expulsion cannot be prevented; but where the discharge precedes the pain, if the child be alive, it frequently may." Still, however, this will necessarily be modified by the part where the separation has taken place. Thus if it has occurred in the immediate vicinity of the os uteri, the blood readily finds an exit; and thus we shall have a discharge of blood with little or no uterine contraction, and without much danger of expulsion. Where, however, the separation has taken place in or about the fundus, there will be pain, and at first without hemorrhage; by degrees, as the blood finds its way towards the os uteri, more and more of the ovum becomes detached, so that at length the greater portion is separated from the uterus; it becomes as a foreign body, and expulsion soon follows, with more or less hemorrhage. These points have been most ably treated by Dr. Dewees in his compendious system of midwifery, and are well worthy of attention. The temporary increase of congestion in the uterine circulation, which occurs at what in the unimpregnated state would have been a menstrual period, may directly excite expulsion by extravasation of blood between the uterus and ovum, or indirectly cause it by destroying the life of the embryo.

Professor von Siebold remarks that, "where abortion happens during the first weeks of pregnancy, the symptoms produced bear a strong resemblance to those of menorrhagia. These attacks usually occur at the periods when the catamenia were wont to appear: this discharge is very profuse, accompanied with severe pain and mixed with coagula and portions of membrane like decidua. After their expulsion the symptoms of pregnancy cease, and the patient usually recovers very quickly." This fact has been noticed by none of the other authors whose essays we are reviewing, except Dr. Copland; Dr. Burns mentions it, but adds a theoretical explanation, viz. that the ovum has not yet entered the uterus, which we deem wholly unnecessary, as the presence of a very small ovum in the discharge can scarcely be detected. The fact has also been noticed by Dr. Locock in his admirable paper on Dysmenorrhœa in the Cyclopædia of Practical Medicine, where abortion at a very early period has been mistaken for unusually severe attacks of dysmenorrhœa. "The degree of hemorrhage is not always in the ratio of the advancement of gestation; though, generally speaking, under similar circumstances, it may be laid down to be the case; for the loss of blood, for the most part, is governed by the period of conception, the force of the remote cause, the degree or extent of detachment, the vigour of the general circulation, the period that elapses before uterine contractions can be successful in the expulsion of the ovum, and the state of the ovum, that is, whether it remains entire or has been forced open so as to expend the liquor amnii." (*Dewees*, p. 90.)

In considering the *treatment* of abortion we decidedly prefer the arrangement made use of by Dr. Copland: he divides it into the preservative, the palliative, and the remedial. To consider this subject fully would require more space than we can afford; we cannot, however, but express our surprise that the observations on this subject by Mr. Charles White, of Manchester, have been so entirely unnoticed by our authors. By no one have the mischievous effects of indiscriminate bloodletting in cases threatening abortion been more clearly pointed out than by this highly practical author. "From the success (says Mr. W.) I have seen attend this practice, (viz. cold bathing,) in preventing miscarriages, and many of the disorders peculiar to the pregnant state, particularly nausea and vomiting, I am satisfied they are much more seldom to be attributed to a plethora than to weak, lax fibres." Although he has not noticed the observations of Mr. White, Dr. Copland has not been unmindful of the facts, and observes that "in every instance the preservative treatment must be based upon our views respecting the pathological state of the uterus, and of the whole frame at the time of prescribing it." His observations at § 32, 33, and 34, are very excellent. Dr. Lee's remarks are equally correct: "where there are no symptoms of local or general plethora and excitement, bloodletting is contra-indicated." Dr. Dewees seems altogether to have overlooked the cases of abortion depending upon a "weak, lax fibre," as described by Mr. White. We cannot agree with him in so general a recommendation of bleeding and strict antiphlogistic regimen, and must in defence of himself quote an excellent observation from his work on Children. "We are warranted by long experience to declare that, unless plethora produce some direct evidence of its mischievous tendencies, as headach, pain in the chest, a sense of fulness in the head upon stooping, giddiness, &c. the patient should not have recourse to bleeding without the express approbation of the physician. To women who are in the habit of miscarrying, this proscription of indiscriminate bleeding is particularly important, especially as it is the remedy almost universally resorted to for its relief, than which in many instances nothing can be more preposterous or improper. We are justified in saying it has very often produced the evil it was intended to prevent." (*Dewees on Children*, § 36 and 37.) Neither can we agree with him in the present article where he says, "the loss of a few ounces of blood is absolutely necessary where there is a painful aching in the back, a heaviness about the loins, a sense of weight and bearing down, even without pain," &c. because cases are not unfrequently met with, where, from great debility and want of tone, these very symptoms are best relieved by bitters, mineral acids, chalybeates, and the cautious exhibition of wine.

Dr. Copland has merely enumerated the various means recommended by different authors for plugging the vagina, without informing us what peculiar form of tampon he himself recommends. We have always preferred the simple sponge of Dr. Dewees as being the nearest imitation of the plug which nature makes use of, viz. a coagulum; it produces less irritation and is introduced and removed with much more facility than the other species of plug. In speaking of the means for exciting the uterus to full contraction Dr. Copland says, " When the embryo only is expelled, the appendages being still retained, or when the hemorrhage is great, the entire ovum still remaining in the uterus, the ergot of rye will often prove of inestimable service, and when given in the form of decoction with as much borax as it will dissolve will seldom disappoint our expectations." (§ 37.) This mention of borax deserves notice. Borax was a remedy many years ago in considerable repute for its efficacy in exciting uterine contractions, and certainly it does appear to possess this power to a considerable degree; the combination of it with ergot will probably render the latter a more certain remedy. In assisting the removal of the ovum from the uterus Dr. Dewees has given a description and drawing of the wire crotchet recommended in his compendious system. The author in the Cyclopædia of Practical Surgery has taken a faithful copy of this instrument, as also (with slight modifications) of the prophylactic treatment recommended by Dr. Dewees.

The *Pince à faux Germe* has been strongly deprecated by Professor Dugès; but he has not mentioned an excellent point of practice recommended by this celebrated accoucheur, viz. of throwing up a powerful stream of tepid water into the vagina to dislodge and bring away the ovum.

Several other points of interest present themselves in a comparative view of these different memoirs, but our limits forbid extending this notice any further. We consider the articles by Drs. Copland and Lee as by far the best. The first is a most valuable digest of the best information upon the subject, and contains almost every thing which is necessary to be known upon it, arranged with much simplicity of method and given in a clear perspicuous style. The article of M. Dugès is also of considerable merit, but it is very far from being complete; nor are the rules for treatment laid down in that clear and simple manner which we so admire in the above-mentioned essays. From Dr. Dewees we confess to have expected a first-rate article, because the excellent observations which have already emanated from his pen warranted such a hope, and we can only regret that he has not devoted more space to the consideration of so important a subject. Dr. Ryan's article is below the present level of our science and our literature.

---

